# Overcoming challenges in glioblastoma treatment: targeting infiltrating cancer cells and harnessing the tumor microenvironment

**DOI:** 10.3389/fncel.2023.1327621

**Published:** 2023-12-21

**Authors:** Mario Chiariello, Giovanni Inzalaco, Virginia Barone, Lisa Gherardini

**Affiliations:** ^1^Institute of Clinical Physiology, Consiglio Nazionale delle Ricerche, Via Fiorentina, Siena, Italy; ^2^Core Research Laboratory (CRL), Istituto per lo Studio, la Prevenzione e la Rete Oncologica (ISPRO), Via Fiorentina, Siena, Italy; ^3^Department of Medical Biotechnologies, University of Siena, Siena, Italy; ^4^Department of Molecular and Developmental Medicine, University of Siena, Siena, Italy

**Keywords:** glioblastoma, local delivery, microenvironment, blood brain barrier, drug repurposing

## Abstract

Glioblastoma (GB) is a highly malignant primary brain tumor with limited treatment options and poor prognosis. Despite current treatment approaches, including surgical resection, radiation therapy, and chemotherapy with temozolomide (TMZ), GB remains mostly incurable due to its invasive growth pattern, limited drug penetration beyond the blood-brain barrier (BBB), and resistance to conventional therapies. One of the main challenges in GB treatment is effectively eliminating infiltrating cancer cells that remain in the brain parenchyma after primary tumor resection. We’ve reviewed the most recent challenges and surveyed the potential strategies aimed at enhancing local treatment outcomes.

## Graphical Abstract

Current treatment approaches for glioblastoma and glioblastoma recurrences. 
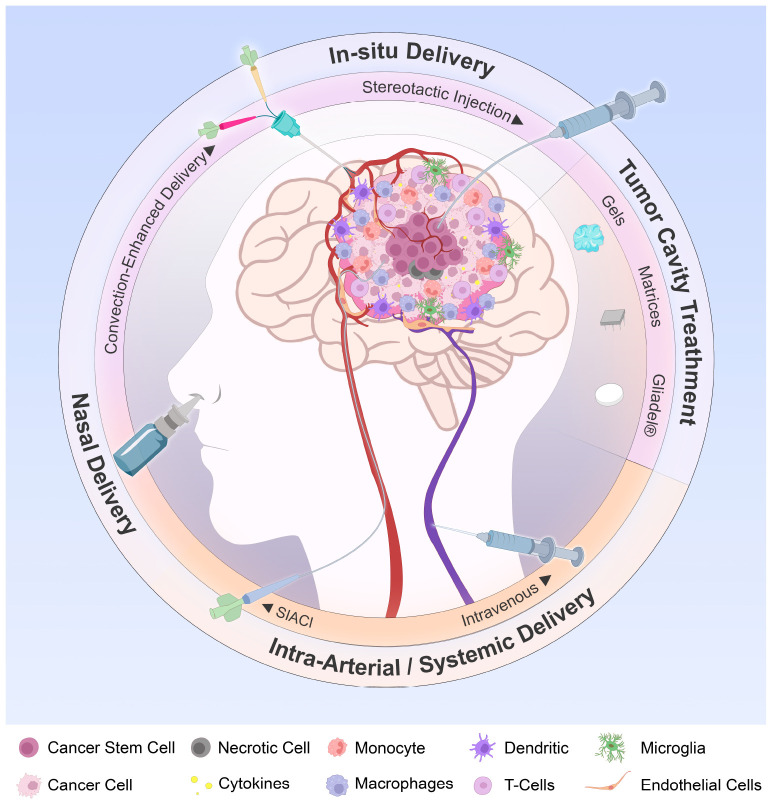

## Introduction

Glioblastoma is the most prevalent malignant primary brain tumor and recent scientific and technical advances have allowed for a deeper understanding of the etiologic relevance of the heterogeneity of GB. Based on the current classification of the tumors of the central nervous system (CNS), among adult-type diffuse gliomas, astrocytic tumors without mutations in the Isocitrate dehydrogenase (IDH) genes are termed glioblastomas IDH-wildtype (Torp et al., 2022). The presence of microvascular proliferation and/or necrosis and at least one molecular alteration identified as predictive of tumor aggressiveness, such as Epithelial Growth Factor Receptor (EGFR) amplification, Telomerase promoter (TERTp) mutations (Brás et al., 2023) up-regulation of chromosome 7 and loss of chromosome 10 (Zhao et al., 2023) allows for a diagnosis of glioblastoma IDH-wildtype CNS WHO grade 4 (since now, GB).

Despite all the innovation that generates the “holistic representation of the evolving disease” (Asleh et al., 2023), GB remains mostly incurable, with a median survival of 14 months and a 5-year survival rate of 5.7%, primarily due to its challenging characteristics: (i) invasive growth pattern, making complete surgical resection nearly impossible; (ii) localization beyond the blood-brain barrier (BBB), which severely limits drug penetration; (iii) resistance to conventional radiotherapy and chemotherapy (Grochans et al., 2022).

Indeed, GB is strongly refractory to most anti-tumor treatments, one of the main reasons lying in the difficulties of tackling its cellular heterogeneity. In the tumor, in fact, the cell population is represented by differentiated GB cells, stem-like cells (GSCs) as well as by some elements of the microenvironment milieu among which endothelial cells, vascular pericytes, macrophages, and other types of immune cells (Zhao et al., 2023). Sequencing technology advances and transcriptional stratification has further corroborated the complexity of this picture contributing to further subdivide GB in proneural, classical and mesenchymal subtypes. In this scenario, the developmental dynamicity of GSCs add another layer of complexity to the heterogeneous nature of the tumors. GSCs are indeed considered at the top of hierarchic lineage of cells holding stem cell-like regeneration ability (Lathia et al., 2015) but share several common molecular markers with normal adult neural stem cells and progenitor cells, originating some ambiguity regarding their definition and identification (Zhang G. et al., 2023). Recently, single-cell RNA sequencing (scRNA-seq) on patient derived samples suggest that GBM cells do not exist as separate populations but rather evolve from stemness to differentiation (Yan et al., 2023). These findings are hardly good news, as GSCs have been found to be deeply implicated in tumor progression, drug resistance and tumor recurrence (Guo et al., 2023). The attention of researchers therefore turns to this class of cells and their plastic nature in search for new regulatory protein to be pharmacologically targeted as Achilles’ heel of tumor cells survival. In this scenario, new homeostatic regulatory proteins were identified: for example, the ZIP14 (SLC39A14) protein, which mediates the cellular uptake of manganese, iron and zinc, has been indicated as a possible mediator of cellular ferroptosis-related cell death (Zhao et al., 2022; Zhang Y. et al., 2023). Similarly, the chloride intracellular channel-1 (CLIC1) is known to be instrumental for tumor proliferation in several solid tumor including GB and his overexpression on GSCs is inversely associated with patient survival (Setti et al., 2013), suggesting CLIC1 to be a potential target and prognostic biomarker (Randhawa and Jahani-Asl, 2023). Accordingly, a novel class of biguanide-based derivatives used as CLIC1-inhibitors has been recently developed and holds promises for the treatment of CLIC1-expressing glioblastomas (Barbieri et al., 2022).

Interestingly, the presence of stem cells in the bulk of glioblastomas has been also strongly associated to cancer multidrug resistance (MDR) (Mattei et al., 2021), which remains a serious challenge in GB therapy as it seriously limits the effects of different chemotherapeutic drugs (Tian et al., 2023). Indeed, as MDR has been often associated with the expression of p-glycoprotein (p-gp), an ATP-binding cassette (ABC) transporter that promotes efflux of chemotherapeutics from tumor cells, this protein has been recently suggested as a druggable therapeutic target (Hasan et al., 2023). Indeed, the p-gp inhibitor reversan, in association with magnetic nanoparticle-mediated hyperthermia, was able to increase the cytotoxic effect of Doxorubicine treatment to eliminate bulk tumors along with the GSC population (Hasan et al., 2023).

Mounting evidence has also been gathered to clarify the role of proteins of the tumor micro-environment (TME) mediating the regulation of cell adhesion and migration (Caverzán et al., 2023; Khan et al., 2023). In this view, TME becomes the scenario of the dynamic dialogue between tumor cells and tumor infiltrating immune system elements, mostly tumor-associated macrophages (TAMs), monocytes, T cells and resident microglia (Eisenbarth and Wang, 2023). TAMs indeed provide a major contribution to cancer growth and immunosuppression, causing resistance even to the most effective immunotherapies, ultimately paving the way to tumor recurrences (Zhang L. et al., 2023). Moreover, in aggressive brain tumors, TME host tumor associated hyper-vascularization. Indeed, the malfunctioning of the tight junctions in the endothelial cells of the tumor associated vascularization may favor the buildup of fluid in the tumor district leading to edema and increased intracranial pressure, partially restored with steroid anti-inflammatory therapy (Cenciarini et al., 2019).

Blood-Brain Barrier’s pericyte’s disruption and hypoxia sustain small vessel proliferation and, in the small vessels district, the dialogue between cancer cells proteins such Nestin and CD133 and endothelial biomarkers such as CD34 proteins facilitates brain metastasis, sustaining tumor cells circulation and subsequent tissue infiltration (Schiffer et al., 2018). More recently, the development of vascularization in GB was also described using genomic analytical tools, to create a risk prediction model for GB, where prognostic differentially expressed angiogenesis-related genes (PDEARGs) become potentially druggable prognostic biomarkers of regulatory networks and provide valuable insight to tackle the role of neovascularization in these tumors (Wan et al., 2023).

Recurrent tumors also exhibit changes in the microenvironment composition, characterized by higher levels of tumor-infiltrating lymphocytes (TILs), macrophages, and increased expression of Programmed Death-Ligand1 (PD-L1) and Programmed Cell Death Protein1 (PD-1) compared to primary tumors (Karschnia et al., 2023; White et al., 2023). This suggests a strong relationship between tumor heterogeneity, immune system involvement in recurrences (Hoogstrate et al., 2023; White et al., 2023) and supports the potential use of peritumoral microenvironmental markers for patient stratification (Riahi Samani et al., 2023). Furthermore, these findings may drive the development of novel precision immunotherapeutic tools (Pornnoppadol et al., 2023; White et al., 2023).

Importantly, a key challenge in GB treatment is effectively targeting and eliminating infiltrating cancer cells that persist in the brain parenchyma after primary tumor resection. Current treatment approaches involve a complex combination of surgical resection, radiation therapy, and concurrent chemotherapy using temozolomide (TMZ) (Fisher and Adamson, 2021). However, the anti-tumor efficacy of TMZ is significantly hindered by its limited ability to cross the BBB (reaching only 20% of blood concentration) (Grochans et al., 2022) and various cellular mechanisms conferring therapeutic resistance. Following the surgical removal of the primary tumor, a lower residual tumor volume is positively correlated with a more favorable prognosis. In this context, it is crucial to identify the microscopic tumor margins hidden within the brain parenchyma and prevent loco-regional recurrence (Bütof et al., 2022). Thus, the quest is to develop effective treatments capable of intercepting residual cells, possibly tumor stem cells, beyond the BBB while potentially countering the activity of favorable microenvironmental elements sustaining tumor invasiveness (Garcia-Diaz et al., 2023).

Here, we aimed at highlighting various strategies employed to overcome the challenges posed by the BBB. Nanomaterial-based delivery systems will be discussed, emphasizing their ability to enhance drug penetration into the brain and improve treatment efficacy and the use of nanomaterials loaded with single drugs or combination therapies. The focus will be centered on how these approaches can enhance the therapeutic effect by targeting multiple pathways or mechanisms involved in tumor growth and progression. Moreover, the review will delve into targeting druggable microenvironmental factors that contribute to the development of recurrent tumors. Finally, we will explore the potential of local drug delivery strategies for drug repurposing will be examined. Understanding and targeting these factors may help preventing the growth of residual cancer cells and reduce the risk of recurrence.

## Overcoming the BBB

The BBB poses a significant challenge for the systemic delivery of chemotherapeutic agents to treat intracranial diseases. It is composed of endothelial cells, pericytes, astrocytes, microglia, and neurons, forming a structural barrier that restricts the passage of molecules into the brain (Sprowls et al., 2019). Endothelial cells lining the brain’s capillaries express tight junctions that seal the gaps between adjacent cells, preventing the free movement of molecules between the blood and the brain. Additionally, these cells lack fenestrations and pinocytic activity, further impeding the passage of substances. Pericytes, closely associated with endothelial cells, regulate capillary diameter and cerebral blood flow while supporting microvascular stability and the BBB’s structural integrity. Astrocytes play a crucial role in maintaining the barrier function through their foot processes, which establish a direct interface between vascular and neuroglial compartments.

Under normal physiological conditions, the BBB’s specific ion channels and transporters maintain an optimal microenvironment for brain function, regulating the levels of neurotransmitters, controlling ionic homeostasis, and protecting against neurotoxins. Water-soluble nutrients, metabolites and small lipid-soluble molecules can passively diffuse through the BBB and enter the brain (Kadry et al., 2020).

Blood-Brain Barrier’s tight junctions and membrane transporters stringently control the exchange of molecules and ions between the blood and the brain. Limited penetration is observed for small lipophilic molecules or those agents that can actively enter by binding to carrier proteins such as transferrin receptor (TfR), epidermal growth factor receptor (EGFR), glucose, or immunoglobulins (Triguero et al., 1989; Arora et al., 2020; Cui et al., 2023; Pornnoppadol et al., 2023).

## Concentrating systemic treatment at tumor site

**FIGURE 1:**
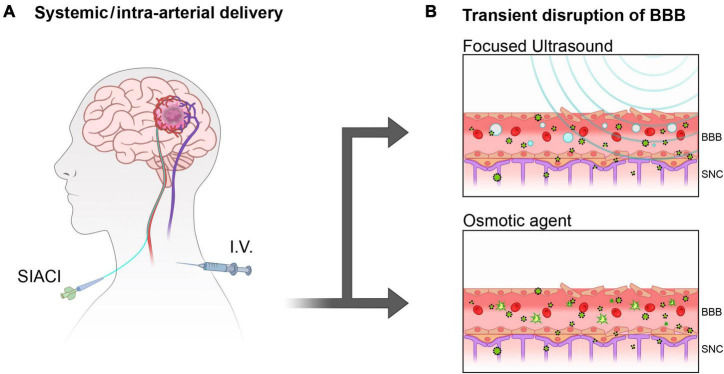
Overcoming BBB with systemically delivered therapies. **(A)** Superselective Intra-Arterial Cerebral Infusion (SIACI) and intravenous injection (I.V) are often administered in conjunction with by temporary opening of the BBB to facilitate delivery to the brain. **(B)** Focused ultrasound (FUS) and Osmotic forces are little invasive methods to temporary disrupt the tightly ordered pavement of the blood vessels constituting the BBB allowing blood-brain exchanges.

Traditionally, the possibility of overcoming the BBB to concentrate systemically administered treatments (Thombre et al., 2023) and diagnostic tracers into the brain (Bae et al., 2023; Figure 1A) is achieved through temporary opening and partial disruption of tight junctions by physical interaction of focused high/low-frequency ultrasound (FUS) (Johansen et al., 2023; Mehta et al., 2023) as well as by using osmotic gradients (Cosolo et al., 1989; Figure 1B). Less conventional methods also include the use of isoflurane (Noorani et al., 2023) to increase the permeability of the brain to small hydrophilic molecules.

Extracranial magnetic resonance imaging (MRI) guided application of focused ultrasounds (FUS) in combination with circulating microbubbles (MB) has shown promise for the treatment of CNS diseases, including GB. High-intensity focused ultrasound (HIFU) can be used for cell and tissue-specific thermo-ablation, directly targeting GB cells (Johansen et al., 2023).

Low-intensity FUS has been FDA-approved for the treatment of untreatable tremors in Parkinson’s disease (NCT03608553). This technique can ameliorate the symptoms in patients. On the other hand, the use of low-intensity FUS combined with circulating MB can temporarily open the BBB (FUS-BBBO) enabling the delivery of anticancer molecules into the brain that may have been ineffective when administered systemically. For example, the intracranial concentration of Panobinostat, administered intra-peritoneally in combination with ultrasound/Magnetic Resonance Imaging (FUS/MRI), was significantly increased compared to sole intraperitoneal injection (Martinez et al., 2023). This led to a significant reduction in tumor volume and extended survival in a patient-derived xenograft (PDX) orthotopic model.

FUS has also been beneficial in the delivery of newly designed conjugates, such as hyaluronic acid (HA) with camptothecin (CPT) and doxorubicin (DOX) (Sun et al., 2022). The flexibility of the polymer in these conjugates facilitates BBB crossing. Similarly, FUS application increased the tumor penetration of a new sensitizer system constituted using a polymeric block, the pH-sensitive polyglutamic acid (PGA) and the chemotherapeutic agent and sonosensitizer DOX, camouflaged with human U87-MG cell membranes administered systemically. This nano-system effectively delivered doxorubicin into U87-MG cells, resulting in tumor growth reduction and overcoming of drug resistance (Chen et al., 2023).

In recent developments, MB-FUS systems are being used actively in drug delivery for cancer immunotherapy. FUS-induced bursting of MB generates an acoustic emission-dependent expression of the proinflammatory marker ICAM-1. Simultaneously, MB-FUS enhances local delivery of anti-PD1 agents and promotes infiltration of T lymphocytes in the tumor microenvironment (Lee et al., 2022).

Other authors combined charged MB with the negatively charged Gambogic Acid (GA) (an active component of a traditional Chinese medicine effective as antiproliferative and tumor infiltration agent) loaded polymeric nanoparticle (GA/PLGA) to form a GA/PLGA-charged MB complex (Dong et al., 2022). Similarly, FUS application benefits the distribution and long-term accumulation of ^89^Zr labeled/cetuximab, an anti EGFR antibody in a preclinical mouse model (Sacks et al., 2018).

In an innovative strategy, intracranial drug depots for small molecules were implanted in mice using bio-orthogonal click chemistry technology, which exploits extracellular matrix molecules to anchor tissue-reactive anchoring of click groups (TRAC). FUS and MB played a crucial role in enabling the non-invasive loading of these drug depots, allowing prolonged and spatially controlled treatment (Moody et al., 2023).

A phase 2 clinical trial (NCT03744026) has been designed for the use of a low intensity pulsed ultrasound (LIPU) device implanted into the skull of patients at the time of primary tumor surgical removal. A different trial (NCT04528680) combined LIPU with injected MB to efficiently increase the delivery of systemically infused albumin-linked paclitaxel in patients with recurrent glioblastoma. LIPU-MB is also being tested in combination with other therapies, such as albumin-bound paclitaxel and carboplatin (Sonabend et al., 2023).

Overall, the application of FUS in combination with MB holds great potential for the treatment of CNS diseases, including glioblastoma. It allows for targeted treatment, enhanced drug delivery, and potential synergies with immunotherapy approaches.

## *In situ* delivery of treatment

**FIGURE 2:**
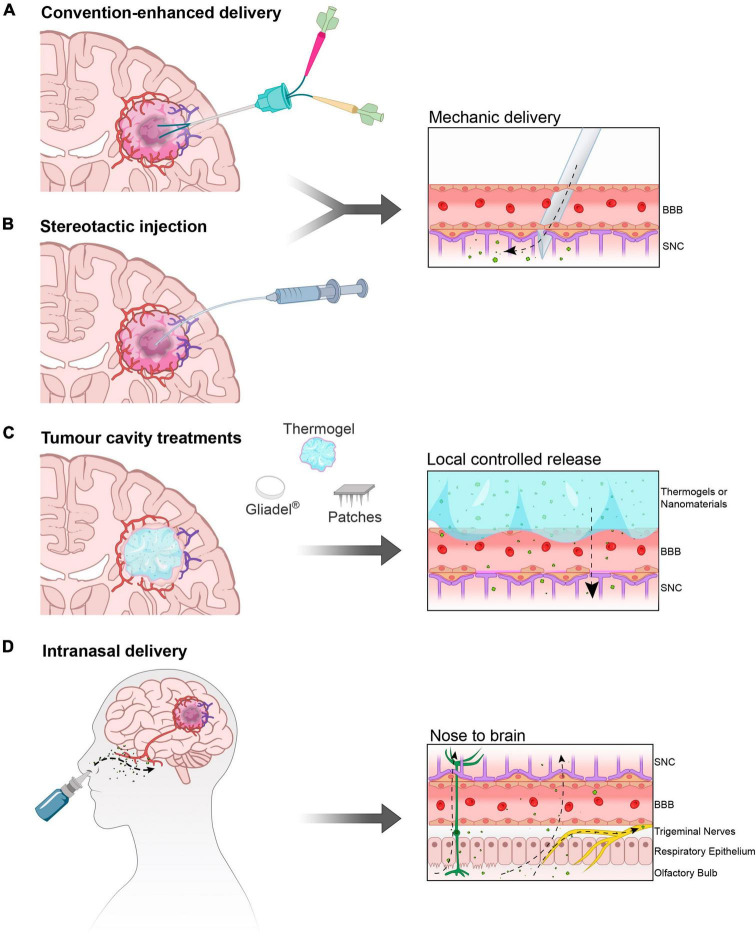
*In situ* and intranasal delivery. **(A)** Convection enhanced delivery (CED) and **(B)** injection of therapy solutions/gel directly into the parenchyma normally after primary tumor resection. **(C)** Deposition of matrices (solid polymers and gels) and mini-devices (micro-needles on patches) in direct contact with the after-resection cavity borders. **(D)** The intranasal route is a portal to the Central Nervous System as therapies inhaled or instilled on the nasal mucosa can bypass the BBB travelling through the olfactory neurons and trigeminal nerves, effectively reaching the Olfactory Bulb and from here different areas of the brain.

Maximizing the safe resection of tumor mass upon diagnosis is crucial for treatment, as the extent of residual volume directly correlates with patient survival. However, even after resection, there are often residual cells hidden within the uneven margins of the resected area, which can lead to tumor recurrence over time. *In situ* post peri-surgery applications aim to concentrate treatments to maximize the chances of killing these remaining cells. However, the presence of surrounding neurons necessitates minimizing functional deficits (Figure 2).

Currently, *in situ* delivery is obtained by convection-enhanced delivery (CED) (Sperring et al., 2023; Thompson et al., 2023) (Clinical Trial: NCT03043391) achieved by gently pushing drugs into the tumor (Figure 2A). Similarly, the intra-arterial delivery of chemicals, with or without the assistance of osmotic gradient (Chu et al., 2022; Wang et al., 2023) can result in effective intracranial treatment. However, the deposition of biocompatible drug-enriched injectable matrices (Figure 2B) that locally release active treatments seems to be the most promising strategy for intracranial controlled release. When resection is possible, patients are surgically implanted with a polymeric matrix for the semi-controlled release of carmustine, the FDA-approved Gliadel^®^. However, this implant has a degree of rigidity that limits its use. With the development of innovative biomaterials such as smart materials, polymeric matrices and hydrogels it will be possible to design more adaptable materials able to adhere to the surgical resection margins of the cavity (Gu et al., 2022; Figure 2C).

Many of these formulations can incorporate nanomaterials that are sensitive to environmental stimuli, including temperature (such as thermogels) (Gu et al., 2022), chemical stimuli such as acidity/CO_2_ (Younis et al., 2023), reactive oxygen species (ROS) (Habra et al., 2022), magnetic force, or X-ray irradiation (Yun et al., 2022). Additionally, the customization of different nanoparticles (Idlas et al., 2023) and the loading of various molecules (Lin et al., 2023) allow for unlimited potential therapeutic combinations. In some cases, nanoparticles loaded with chemotherapeutics are dispersed into gel matrices (Gu et al., 2022), which function as a shelter to reduce molecule degradation and unwanted cytotoxicity (Erthal et al., 2023). New formulations for *in situ* delivery aim at optimizing the effects of first-generation anti-glioblastoma agents such as carmustine and temozolomide, either alone or in combination, to maximize their curative effects, for example overcoming the resistance to TMZ using the O_6_ alkylguanine DNA alkyltransferase inhibitor, dialdehyde O_6_ benzylguanine (DABG), tested in xenograft (Chu et al., 2023) or intracranially delivered, minimizing peripheral toxicity (Chen et al., 2022; Iturrioz-Rodríguez et al., 2023).

*In situ* delivery also presents an opportunity for targeting the tumor microenvironment and stimulating the tumor-suppressive immune system. Recently, hyaluronic acid (HA) has been utilized as an injectable delivery platform for combinatory treatments. HA-Doxorubicin and HA-CpG, an agonist of Toll-like receptor (Catania et al., 2023), were used to stimulate tumor-associated macrophages (TAM). Similarly, DOX-loaded mesoporous polydopamine (MPDA) nanoparticles were encapsulated in macrophage-derived nanovesicles (M1NVs) and used as immunostimulant effectors, while fibrin hydrogels serving as *in situ* delivery vehicles (Zhang R. et al., 2023).

GB recurrence has been recently modeled, in preclinical settings, by partially resecting the primary tumor. Bianco and coworkers have successfully demonstrated this approach (Bianco et al., 2017; Kubelt et al., 2023), and more recently, a minimally modified technique has been developed (Sun et al., 2023). Live imaging is frequently used to monitor the inevitable reappearance of the intracranial tumor due to residual cells and the presence of glioma stem cells (GSCs). Following resection, the cavity created in the brain tissue provides an opportunity for delivering curative treatments. One promising approach involves injecting a biodegradable thermosensitive triblock copolymer, poly (D,L-lactic acid-co-glycolic acid)-b-poly(ethylene glycol)-b-poly(D, L-lactic acid-co-glycolic acid) (PLGA-PEG-PLGA), that contain the anticancer therapy for sustained local release. These formulations mostly include TMZ (Zhang R. et al., 2023); however combinatorial treatment can be achieve also using less conventional anti-GB agents such as curcumin (Liang et al., 2023). Intra-tumor injection can also be performed with allosteric tissue elements (Figure 2B). In a phase I trial, a suicide gene therapy was attempted by injecting adipose tissue-derived mesenchymal stem cells (ADSCs) carrying the herpes simplex virus-thymidine kinase (HSV-TK) gene into patients with non-surgically removable recurrent GB (Oraee-Yazdani et al., 2023). A notable innovative therapeutic tool carrier has recently been developed (Sun et al., 2022). This carrier consists of allomelanin nanoparticles (AMNPs) loaded with a checkpoint inhibitor, CLP002, and camouflaged with cancer cell membrane coatings (CCM). The delivery system, named AMNP@CLP@CCM, can penetrate the BBB, interfering with immune activity, and sustaining photo-thermal ablation with AMNPs. Ultimately, these combined effects will synergistically kill tumor cells.

The murine model of intracranial resection of primary tumor mass is frequently used for the preclinical investigation of treatment against recurrences, filling the resection cavity with the local therapy. “Smart” hydrogels, such as chitosan-based thermogels enriched with SiO_2_-TMZ loaded nanoparticles, have proven effective in reducing recurrences in a U87-MG recurrence model. The use of silica particles as carriers represents an unconventional but equally effective choice compared to the more commonly used PCL-TMZ loaded nanoparticles (Gherardini et al., 2023). Similarly, photosensitive polymeric matrices have been previously employed to treat recurrences with paclitaxel (PTX) (Zhao et al., 2018). A novel approach to target residual cells in GB involved the use of polymeric microneedle patches designed to anchor within the irregular margin. These patches were loaded with polymer-coated nanoparticles containing either cannabidiol (CBD) or olaparib (OLA). The uniqueness of the study lies in both the choice of the carriers and the therapeutic agents (Muresan et al., 2023). OLA, a PARP inhibitor, has been utilized in combination with TMZ in both preclinical studies (Zampieri et al., 2021) and clinical trials (Lesueur et al., 2019). However, there are only a few encouraging reports on the use of CBD, the non-psychotropic component of cannabis, showing tumor growth reduction (Lah et al., 2022) and increased survival rates in glioblastoma patients (Likar et al., 2021).

## The intranasal route

The intranasal route offers a direct pathway for active molecules to reach the CNS, making it an attractive option for drug delivery to the brain. This route serves two purposes: delivering drugs directly to the brain (nose-to-brain) and increasing peripheral vascular concentrations (nose-to-blood), similarly to pulmonary delivery methods. The olfactory nerve pathways serve as the gateway to the Olfactory Bulb, where drugs deposited on the nasal mucosa can travel through the olfactory neurons and trigeminal nerves, effectively reaching different areas of the brain (Figure 2D).

Through this way, active formulations can avoid first pass (enzymatic degradation and clearance) metabolism, increasing their bioavailability in the CNS, minimizing the occurrence of peripheral side effects. However, it is important to note that local damage of the nasal mucosa and brain tissue can limit the amount of active drugs that can be delivered.

In general, highly lipophilic drugs or nanoparticles with a low molecular weight are well-suited for intranasal delivery strategies. These characteristics enable better penetration and absorption through the nasal mucosa, facilitating efficient transport to the brain.

Recently, the utilization of intranasal routes for treating GB has been reviewed, focusing on the advantages and disadvantages for patients (Goel et al., 2022). Furthermore, in the past decade, the availability of tunable nanoparticles has stimulated multidisciplinary research to design and test various promising formulations (Montegiove et al., 2022). A recently completed clinical trial (NCT04091503) examined the safety and effectiveness of intranasal administration of TMZ in patients with GB and, recently, nanocarriers have been specifically designed for intranasal delivery. For instance, a monoclonal antibody anti-EphA3 was utilized to target TMZ-loaded gold nanoparticles (anti-EphA3-TMZ@GNPsTMZ) for photothermal therapy in nose-to-brain delivery (Yu et al., 2022). Chitosan-coated PLGA nanoparticles were optimized for nose-to-brain delivery of carmustine into the healthy Albino rat brain, resulting in significantly increased chemotherapeutic concentration compared to plasma levels. However, no indication of efficacy against tumor growth *in vivo* was reported (Ahmad et al., 2022).

Decorating nanoparticles with transferrin to achieve targeting of TfR appears to be an efficient approach for intranasal delivery, as already demonstrated (Sandbhor et al., 2023). Lipid nanoparticles (LNPs) were used for the co-delivery of paclitaxel (PTX) and miltefosine (HePc), a proapoptotic agent, resulting in significant tumor reduction and increased survival in mice after intranasal treatment compared to systemic Taxol^®^ and nasal free drug administration. The role of meningeal pathways and of the lymphatic system in intranasal delivery has recently been studied revealing reduced liposomal transport in GB (Semyachkina-Glushkovskaya et al., 2022). The application of biocompatible infrared photo stimulation of meningeal lymphatic vessels in the cribriform plate could potentially enhance flux and delivery. Finally, we highlight few rare examples of nanoparticle tracing after intranasal delivery (Han et al., 2023) mapping their localization in the tumor site, brain parenchyma and more significantly intercepting accumulation in internal organs and overall body districts (NIR and SPECT/CT imaging), which is crucial for targeting and assessing off-target toxicity. Gold nanorods (AuNRs) tested as a platform for delivery in CNS were functionalized by adding the fluorescent dye Cyanine5 (Cy5) for optical imaging or a metal chelator, diethylenetriaminepentaacetic dianhydride (DTPA) hinged by PEG to ^111^Indium for nuclear detection, allowing detection of the brain area distribution and the peripheral organ distribution of the NP after entering the CNS (Han et al., 2023).

In conclusion, the nose-to-brain route holds great potential for repurposing conventional drugs for novel therapeutic applications.

## Drug repurposing

Drug repurposing is a cost-effective strategy for cancer treatment, serving as an alternative to *de novo* drug discovery. In the case of GB, candidate molecules for repurposing are often identified through biomedical and biogenetic profiling of patients and *in silico* docking simulations (McGowan et al., 2023; Roy et al., 2023).

Among these candidates, FDA-approved drugs that are currently used for treating other conditions have shown promise (Jones et al., 2023; Moretti et al., 2023; Murali and Karuppasamy, 2023; Vítovcová et al., 2023). Recent preclinical examples include Flubendazole, an inhibitor of microtubule growth that activates autophagy and STAT3-dependent apoptosis, and auranofin (AF), an inhibitor of TrxR1, used alone or in combination with the prooxidant menadione (Szeliga and Rola, 2022). Hydroquinidine (HQ) is another repurposing candidate (Yavuz and Demircan, 2023) to induce GBM cell death overcoming TMZ resistance that could be a good candidate for *in vivo* study.

Repurposing or repositioning anticancer agents that have demonstrated efficacy *in vitro* but failed to produce therapeutic effects *in vivo*, or those causing unacceptable off-target effects when administered systemically, could find a second life through local delivery. For example, intranasal administration of Temozolomide (TMZ) (Clinical Trial: NCT04091503) or systemic administration of Placlitaxel, in combination with albumin to induce osmotic opening of BBB (NCT04528680) is under investigation, as well as the intracranial deposition of Paclitaxel in OncoGel^®^ NCT00479765 (Morales and Mousa, 2022) to elicit a stronger antitumor effect. A single dose of intra-arterial mannitol should facilitate the temporary opening of the BBB to allow superselective intra-arterial cerebral infusion (SIACI) (Figure 1A) of a high single dose of TMZ (up to 250mg/m2) to achieve a more efficient tumor site targeting (NCT01180816). Similarly, SIACI protocol with mannose osmotic pretreatment is attempted with Cetuximab (CTX), targeting the Epidermal Growth Factor Receptor (EGFR) is under study in a Phase I clinical trial to treat recurrent GB (Clinical Trial: NCT02861898).

## Conclusion

Recent advances *in loco*-regional treatment in GB focused on overcoming the BBB and targeting microenvironmental proteins to enhance therapeutic efficacy. “The Holy Grail,” “the Magic Bullet,” “the Trojan Horse”: these are all metaphors that can be used to define the goal to find a mythic nano-devise or a molecular tool that could be able to overcome the BBB and deliver its anticancer therapy for an effective personalized treatments to benefit GB patients. On the other hand, strategies such as focused ultrasounds with microbubbles, intracranial drug depots, and *in situ* delivery using nanomaterials and biomaterials show promise in improving drug delivery combating GB recurrence and improving patient’s overall survival and quality of life.

## Author contributions

MC: Conceptualization, Data curation, Funding acquisition, Project administration, Writing−original draft. GI: Conceptualization, Data curation, Methodology, Software, Writing−original draft. VB: Conceptualization, Data curation, Writing−original draft. LG: Conceptualization, Data curation, Methodology, Writing−original draft, Writing−review and editing.
